# Enteroclysis in the Various Forms of Enterocolitis and the Zymotics Generally1Read by title at the twenty-seventh annual meeting of the Medical Association of Central New York, Buffalo, N. Y., October 16, 1894.

**Published:** 1895-06

**Authors:** F. W. Bartlett

**Affiliations:** Buffalo, N. Y.


### ENTEROCLYSIS IN THE VARIOUS FORMS OF ENTERO-COLITIS AND THE ZYMOTICS GENERALLY.[1]

By F. W. BARTLETT, M. D., Buffalo, N. Y.

"FAMILIAR in our ears as household words," will be the comment of my local brethren, as it is now past eleven years since I reported to the then Buffalo medical and surgical association, now the academy, my first cases of enteroclysis. They were admitted by all to be original work ; not but that enemas of various forms are as ancient as medical history, but the substance used for the purpose as a germicide, hydrarg. bichlorid in defined quantity and solution, was novel in their experience. For several years this treatment was limited to dysentery, as that term is generally understood, but soon, influenced by success and by a belief that other forms of disease had a similar causation in the invasion of special germs, I added to my repertoire typhoid fever; then, step by step, all forms of diarrhea and lastly of the zymotics generally.

It is now my purpose to specially define my mode of operation, which I consider of particular importance. It is not usually so necessary in ordering enemas to give instruction, but, in my observation, the treatment of colitis by active germicides should be thoughtfully considered. I pass for exhibition the apparatus employed, as also a sample of the rectal tubes of former and even the present times. It is, in my estimation, a dangerous and wholly unnecessary instrument. The sigmoid is not so easily passed and fatal traumatisms may follow rude manipulations. It is only necessary, by position of the patient, to convert the descending colon to a horizontal direction and, in doing so, to change the transverse colon to a descending one. The introduction of the catheter for any special distance is unimportant. If it readily enters entirely it is well, otherwise the enema will flow promptly to the transverse colon, now become a gravitating canal and so on to the iliocecum and often to the ilium. Now, my solution never exceeds one grain bichloride to 8,000 grains (one pint) of warm water. I employ, usually, four pints and I use for the reservoir a glass or glazed vessel, such as a two-quart glass pitcher or fruit jar, and locate myself and patient as follows : first arranging suitable protectives for the mattress, place the patient on his *right* side, with pelvis rather above than below the shoulder level. Bring the pelvis to the edge and rather over the edge of the mattress. I place one

1. Read by title at the twenty-seventh annual meeting of the Medical Association of Central New York, Buffalo, N. Y., October 16, 1894.

common chair opposite the sacrum used for the reservoir and one for myself to the left and near the nates. I then drop the suction end of the syringe to the bottom of the reservoir, introducing by the left hand the oiled catheter within the sphincter, and try, by gentle manipulations, to urge it forward, but do not risk spasm of the tender rectal surface by persistence. I pump, by the right hand, steadily and gently, and usually find the catheter can be passed farther upward. As soon as the reservoir is emptied, place the patient upon an ordinary slop jar or chamber pail, in preference to the usual utensil, thus securing full capacity for the dejection. The enema will be promptly and noisily ejected. After reasonable time return the patient to bed, lay him on left side to favor gravitation of retained fluid, and place sufficient removable material under him to protect the mattress. In a few minutes expect further use for the jar and treat subsequent pain or restlessness *secundem artem.* You may readily control dysentery in the adult by one enema, rarely more than two will be required, repeating second or third day, if needed. If the patient is placed upon the left side in operating, you must overcome the pressure of the injected fluid as it rises in the now vertical transverse colon, also the spasmodic action of same due to the wholly unnecessary pressure upon its walls. Before the enema passes to the ascending colon, the tension may become unbearable and compel you to desist, defeating the operation.

As is most proper, we avail ourselves, as speedily as possible, of the recent bacterial discoveries, which will be fully described at this meeting. "The people who walked in darkness saw a great light." We are informed as to germ causation, and, as physicians, we should profit by the revelations of science. Knowing something of vital powers of resistance, we must, if unable to diffuse antitoxins in the circulation, inhibit the development of germs and consequent ptomaines and toxines by efforts for their destruction while still within the alimentary canal. Work by safe germicides in the stomach and ileum and by thorough flushings, always of germicide qualities, in the colon. Remove the retained secretions of diphtheria, scarlet fever, measles, and the like, from their habitat in the intestines.

I have had no relapses in dysentery or typhoid, and if in the latter disease there has occurred, after the fifteenth day, any special rise in temperature, it was induced by some extraneous cause. In infantile cases, one grain bichloride to 16,000 or

20,000 has been my rule, using one or two pints this strength, repeated *pro re nata.* While in adult cases I insist on the patient retaining the full enema, two quarts, such coöperation is not expected from young children. I introduce the whole catheter, if easily done, to compensate for premature expulsion of enema.

Experience in this plan of treatment is not limited to myself in this city. I have reason to assert that it has found favor with many physicians, often with my coöperation. It is no exaggeration to say that I have administered, in the eleven years now closed, nearly one thousand germicide enemas. I have limited the germicide used to one grain bichloride to 8,000 to 20,000 solution. When I have occasionally used warm water only, it has proved useless. The way is open to trial of other and perhaps safer germicides ; open, in fact, to gaseous inflations.

The fierce phlebotomy of Brousais, the alcohol of Stokes, the expectancy of Gull and the semi-freezing of Brand may be safely superseded by anticipatory germicide treatment. In typhoid make this your very first treatment. It has claims almost specific, particularly if your patient has hot water sponging every fourth hour. Avoid cold as conducive to flatulent colic, local congestions, diarrhea, hemorrhage, wakefulness, spasms, and the like. Control temperature by antipyretics and purely abdominal ice-packs, if needed. Never let your patient shiver from exposure.

Lacking the advantages of hospital clinical opportunity, I can only offer twenty-seven consecutive successful cases of typhoid in proof of the usefulness of the treatment directed. I am striving to be concise and will only further say, treat all your cases of intestinal fluxes, from the month old infant to the octogenarian, in this way. Flush, also, your scarlet fever, diphtheria and intermittent fever cases, if diarrhea is a prominent symptom. Escape relapses and other sequelæ by prompt expulsion of germs, ptomaines and toxines from the colon.

I would claim originality for this modification in treatment, antedating as it did, in its entirety, any verbal or written communication to the profession.

NOTE.—The syringe preferred is the "Alpha," on account of exit tube favoring easy attachment of the catheter, by simple insertion, also the continuous current. Any syringe with inserted tubes will answer. An ordinary No. 10 (American) catheter makes good connection by cutting off bone finish. Insert cut end.

